# Interaction between hypotension and age on adrenal crisis diagnosis

**DOI:** 10.1002/edm2.205

**Published:** 2020-12-02

**Authors:** R. Louise Rushworth, Thomas Goubar, Cecilia Ostman, Shaun McGrath, David J. Torpy

**Affiliations:** ^1^ School of Medicine, Sydney The University of Notre Dame Darlinghurst NSW Australia; ^2^ John Hunter Hospital New Lambton NSW Australia; ^3^ Endocrine and Metabolic Unit Royal Adelaide Hospital and University of Adelaide Adelaide SA Australia

**Keywords:** adrenal crisis, adrenal insufficiency, hypotension

## Abstract

**Objective:**

To determine whether adrenal crisis (AC) identification may be affected by the definition of hypotension.

**Context:**

Delays in AC diagnosis can result in adverse outcomes. AC‐related cardiovascular compromise may vary according to baseline blood pressure and may be associated with delayed AC detection in some patients.

**Design:**

A retrospective study of paired systolic blood pressure (sBP) measurements in hospitalized patients with primary AI (PAI).

**Patients:**

Patients with PAI and an acute illness admitted for urgent treatment between 2000 and 2017.

**Measurements:**

A comparison between sBP on hospital arrival and on discharge. Hypotension was classified as either absolute hypotension (sBP 100mg or lower) or relative hypotension (sBP over 100 mg but at least 20 mm Hg lower than discharge sBP).

**Results:**

Of 152 admissions with paired blood pressure measurements, 46 (30.3%) included a medically diagnosed AC. Absolute hypotension was found in 38 (25.0%) records, and a further 21 (13.8%) patients were classified as having relative hypotension. Patients aged 65 years and older had the lowest (14.8%, n = 8) proportion with absolute hypotension but the highest (27.8%, n = 15) with relative hypotension. Use of either absolute or relative hypotension as the criterion for AC diagnosis increased the proportion of patients with an AC by 28.3% and the proportion of patients with an AC in the oldest age group by 130%.

**Conclusions:**

Failure to detect cardiovascular compromise is common in older AI patients, may underestimate the AC rate in this group, and delay essential treatment. Relative hypotension may play a role in AC diagnosis.

## Abstract 2

Differences in presentation and discharge blood pressure were assessed in a sample of patients with primary adrenal insufficiency and an acute medical illness. Hypotension (sBP < 100 mm Hg) on arrival was more common in younger patients while relative hypotension (discharge sBP > 20 mm Hg higher than the presenting sBP) was more common in older patients. The results of this exploratory study suggest that hypotension associated with an adrenal crisis may be missed in older patients if relative hypotension is not assessed in an unwell older patient with adrenal insufficiency.

## 1 INTRODUCTION

All patients with adrenal insufficiency (AI) are at risk of an adrenal crisis (AC) during periods of stress, most commonly an infection.^1^, ^2^ These episodes, which are characterized by hypotension and other symptoms and signs, including confusion, acute abdominal symptoms, and electrolyte abnormalities, have an incidence of between 5 and 8 ACs/100 patient years in treated AI^2^, ^3^, ^4^, ^5^ and appear to have been increasing in frequency over recent years.^6^

Misclassification of AC episodes is a common problem that may affect the care of patients with AI and introduces biases into AI/AC research. Adrenal insufficiency comprises a range of symptoms which are generally nonspecific across many systems and may be confused with the inciting acute illness (eg gastroenteritis vs the nausea and vomiting of cortisol deficiency). Diagnosis of AI, therefore, is based on context and symptom pattern recognition.^2^, ^4^, ^5^ Biochemical features, although quite frequent, are not required for diagnosis.^2^, ^4^, ^5^ In contrast, an AC includes all these features and hypotension, with a caveat requiring a prompt response to treatment to strengthen diagnostic specificity.^2^, ^4^, ^5^ Clinically missed AC events, which often follow a period of symptomatic AI, may be fatal or lead to prolonged morbidity.^7^, ^8^

Of considerable importance in the management of patients with AI is the failure to detect cardiovascular compromise in an unwell AI patient before irreversible circulatory collapse develops. A threshold of systolic blood pressure (sBP) of 100 mm Hg or below has been recommended to diagnose an AC.^2^, ^4^, ^9^ However, for many patients whose usual blood pressure is in or near the hypertensive range, cardiovascular decompensation may be present at a higher sBP than 100 mm Hg. For this reason, the criterion of relative hypotension (which has a suggested definition of: a sBP above 100 mm Hg but at least 20 mm Hg lower than the usual sBP for the patient) was introduced into the AC definition to capture AI patients whose typical sBP is in the hypertensive range but who are unable to maintain that blood pressure in the context of an acute illness.^2^, ^3^, ^9^ Given the general rise in blood pressure with age, this phenomenon is particularly likely in older AI patients, whose risk of poorer outcomes is increased by both the under‐recognition of hypotension and the greater likelihood of other significant comorbid conditions that accrue with age.^2^, ^9^

While the need for the detection of relative hypotension as a criterion for AC diagnosis has been suggested as a useful guide in AI patient management, particularly among older patients, the effect of incorporation of this measurement on the estimation of AC occurrence, including AC incidence estimates according to age, has not been explored. This study investigates this phenomenon by examining differences in sBP measurements, pre‐ and post‐treatment for AI/AC, in a population‐based sample of patients with primary AI (PAI) who were admitted to hospital.

## 2 METHODS

The design of the study and the description of the PAI patient population have been reported previously.^10^ Briefly, the study comprised a retrospective audit of the medical records of patients with at least one admission to a large regional hospital in which there was a principal or comorbid diagnosis of PAI.^10^ For the present analysis, all patients included in the sample had PAI, were on replacement therapy and had been admitted to hospital between 2000 and 2017 for the treatment of an acute medical illness. Patients attending hospital following trauma or for a surgical problem were excluded. In addition, to remove the possibility of other causes of hypotension being confused with or confounding the relationship between illness and AC‐related hypotension, patients with a diagnosis of sepsis, haemorrhage or a principal diagnosis of a cardiac problem were excluded.

Each patient's sBP was measured on arrival (before treatment) at hospital, and this was used as the presentation sBP value for AC assessment purposes. While the patient's usual sBP in the community was unknown, the last sBP measurement prior to discharge from hospital was taken as the best approximation for that patient's baseline blood pressure, as this was likely to be the most representative of the patient's usual status. All eligible records were re‐examined to locate the last blood pressure assessment prior to discharge from hospital, and only patients whose record contained both sBP measurements were included in the analysis.

In this study, the sBP difference was calculated by subtracting the sBP on presentation to hospital from the ultimate sBP prior to discharge. Absolute hypotension was classified as present, if the presenting sBP was 100 mHg or lower. Relative hypotension on presentation was classified as present if the sBP on first attendance was above 100 mm Hg but was at least 20 mm Hg lower than the sBP at discharge. Age was classified into 18‐39, 40‐64 and 65 and older years age groups. An AC was classified as present if it had been diagnosed by the treating clinician and noted as such in the medical record.

Statistical analysis was conducted using the SPSS software package (SPSS Statistics version 25). Categorical variables were evaluated using *χ* ^2^ or Fisher exact tests. A *P* value of <.05 was considered significant.

The study was approved by the St. Vincent's Hospital, Sydney Human Resource Ethics Committee (HREC), the Hunter New England Health Service HREC, and the HREC of The University of Notre Dame, Australia.

## 3 RESULTS

There were 152 admissions of patients with PAI that met the inclusion criteria of the present study. Of these, 46 (30.3%) included a medically diagnosed AC. The majority (67.1%, n = 102) of the patients were female and the average age was 55.4 (18.2) years (range 18‐90 years). The median length of stay was 3.0 (IQR 1, 5) days. Hyponatraemia (serum sodium less than 135 mmol/L) was found in 48.7% (n = 74) and hyperkalaemia (serum potassium greater than 5.0 mmol/L) in 21.1% (n = 32). The distribution of other relevant clinical details for these patients was reported previously.^10^

Absolute hypotension (sBP 100 mm Hg or lower) was identified in 38 (25.0%) patients on presentation to the emergency department. A further 21 (13.8%) patients were classified retrospectively as having relative hypotension (sBP 20 mm Hg or lower than their discharge sBP). In total, there were 59 (38.8%) patients who were classified as having either form of hypotension on arrival at hospital.

Among the patients with either type of hypotension, 42.6% (n = 26) were found to have a diagnosis of AC in their medical record. Of those with absolute hypotension, 53.8% (n = 21) were classified as having had an AC but only 23.8% (n = 5) of the patients with relative hypotension were given an AC diagnosis during that admission. As a result, the estimated number of hypotensive‐ACs was 28.3% higher than the number of medically diagnosed ACs.

IV hydrocortisone was administered to 80.8% (n = 126) of all the patients in the sample, corresponding to 93.6% (n = 44) of patients diagnosed as having had an AC and 80.0% (n = 48) of patients with any form of hypotension.

### 3.1 Age and hypotension

**Figure 1:**
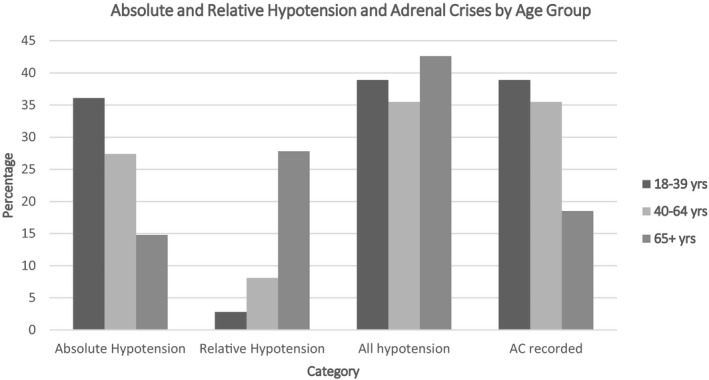
Absolute and Relative Hypotension and Adrenal Crises by Age Group

The distribution of both absolute and relative hypotension varied substantially by age. Absolute hypotension was identified in 36.1% (n = 13) of 18‐39 year olds; 27.4% (n = 17) of those aged 40‐64 years; and only 14.8% (n = 8) of patients aged 65 years or older (*P* = .06). By comparison, relative hypotension was found in 2.8% (n = 1) of the 18‐39 year olds; 8.1% (n = 5) of the 40‐64 year age group; and 27.8% (n = 15) of the oldest age group (*P* < .01) (Figure 1). Consequently, inclusion of relative hypotension increased the overall number of patients who were classified as hypotensive by 29.4% in those aged 40‐64 years and 187.5% in the 65 years and older age group. When both categories of hypotension were combined, the proportion of patients who were hypotensive in each age group was comparable (38.9%, n = 14 in 18‐39 years; 35.5%, n = 22 in 40‐54 years; and 42.6%, n = 23 in the 65 years and older group) (*P* = NS) (Figure 1).

In this sample, a record of a medically diagnosed AC varied by age, being highest (38.9%, n = 14) in the youngest age group, 35.5% (n = 22) in the 40‐64 year age group and lowest (18.5%, n = 10) in the oldest age group (*P* = .06) (Figure 1). However, when both forms of hypotension were chosen as the substitute indicator of an AC, the proportion of hypotensive‐AC patients in each age group was similar. When relative hypotension was included, the proportion of patients in the 65 and over age group who were classified into the hypotensive‐AC category increased the number of ACs in this group by 130%, while the estimates for the two younger age groups remained stable.

## 4 DISCUSSION

The results of the present study demonstrate that, in an ill patient with AI, assessment of cardiovascular compromise using the criterion of absolute hypotension without reference to the patient's usual blood pressure could fail to identify a subgroup of patients whose cardiovascular status is affected adversely, and that this is especially the case in older patients. Consequences of this misidentification may include delays in administration of the emergent therapies, intravenous hydrocortisone and fluid resuscitation, possibly leading to illness progression, a greater likelihood of severe sequelae and even death. Diagnostic misclassification also distorts AC incidence assessments, potentially underestimating the overall incidence of ACs in a population by up to 30%. This phenomenon applies especially in patients aged 65 years and over, in whom the estimated number of cases of AC would have increased by 130%, had hypotension, both absolute and especially relative hypotension, been used as the criterion for assessing the presence of an AC in this patient sample.

Achieving accuracy in AC diagnosis is a pervasive problem in AI/AC research.^2^, ^4^, ^9^ Variability between clinicians in the symptoms and signs that they consider constitute an AC is substantial and makes interpretation of AC rates, and any changes in these, difficult to assess with any certainty.^4^ While, in the present study, the estimated number of ACs, whether clinician‐diagnosed or based on the presence of hypotension, remained the same in the two lowest age categories, the individual patients who were classified as such differed. In contrast, fewer patients in the oldest age group received an AC diagnosis, although considerably more patients were found to have some indication of cardiovascular compromise when relative sBP was included in the assessment. Such misclassification in older age groups would distort the observed relationship between older age and AC risk and may encourage a mistaken belief that AI/AC is less important in older people. The higher prevalence of AI in older relative to younger people also means that comparisons of AC rates between groups may be further confounded by demographic differences, due to the relationship between age and difficulties with AC diagnosis.^9^, ^11^, ^12^, ^13^ Improved AC identification in older patients would not only be of great benefit to research and surveillance of AI/AC; it should also improve the acute and longer term management of individual patients, as the circumstances of each AC should be reviewed with the patient following treatment to identify precipitants and develop strategies that could be used to prevent another episode.^2^, ^4^, ^9^ Further research into the effect of earlier diagnosis of cardiovascular decompensation and the health outcomes of older patients would be of benefit.

Modification of normal values for physiological parameters according to age has been advocated for use in AC/AI diagnosis in children and is well established in paediatric practice generally.^14^ A similar approach to hypotension assessment in adults is physiologically based and would potentially improve the health outcomes of older AI patients, including those with secondary AI, who, while not included in this sample, are also at risk of AC events.^2^, ^9^ Modifications to sBP cut‐off levels in adults have also been advocated in the fields of surgery, emergency medicine, and anaesthetics.^15^, ^16^ These recommendations arise from an understanding that delaying appropriate interventions until absolute hypotension has been established in an older patient would likely have been preceded by vital organ under‐perfusion and confers a greater probability of serious adverse outcomes. Where a patient's usual blood pressure is unknown, typical values for that age or, alternatively, a higher threshold for absolute hypotension in older patients would be suitable replacements.

This exploratory analysis examined data on PAI patients who were part of a larger population‐based study. As the patient's usual blood pressure in an out‐of‐hospital setting was unknown, the last blood pressure measurement prior to discharge was used as the best estimate. While the stress of illness and hospitalization, so called ‘white coat hypertension’, may have exerted an upward effect on the usual sBP in these patients, this would more likely have had a greater effect on the initial blood pressure measurement during the acute, more stressful, phase of the illness than on the estimation prior to discharge.^17^ Resting low sBP in younger individuals may occur but sBP < 100 mm Hg is unlikely in acute illness and we did not observe a difference in AC diagnosis when sBP was not considered as a specific criterion in this age group. Postural change in sBP is assessed in the chronic management of AI patients but is not included in the consensus or other recent AC definitions^1^, ^2^, ^4^, ^5^; has not been well validated in acutely unwell AI patients; and, in this sample, was rarely measured. In addition, AC diagnoses used in this analysis were those that were made by the attending clinician and noted in the medical record and, although diagnostic decisions are subject to variation between practitioners, all patients in this sample were considered sufficiently ill to be admitted to hospital following their acute presentation and the majority were given intravenous hydrocortisone. While intravenous hydrocortisone administration may be associated with some sBP elevation over time (1 week in one study),^18^ this has not been assessed in AI patients who require parenteral treatment to address cortisol deficiency and is unlikely to be reflected in an elevation of the sBP at discharge, given the usually short duration of therapy to manage an AC.^18^ Further, a 20 mm Hg difference compared to usual BP in acute illness has been considered a reasonable cut‐off in several consensus statements although the exact BP reduction for potential AC classification has not been validated against treatment outcomes or prognosis. Similarly, in this analysis we were unable to determine prospectively the effect of assessment of this definition of relative versus absolute hypotension on the earlier use of treatment and prevention of adverse outcomes in affected patients.

In conclusion, the results of this exploratory analysis suggest that identification of relative hypotension could be an important component in the acute management of all ill patients with AI, particularly those in the older age groups. If so, appropriate modifications of diagnostic criteria in the emergency setting should enable affected patients to access appropriate care more promptly, reduce the likelihood of adverse sequelae and improve outcomes among patients with AI.

## CONFLICT OF INTEREST

The authors declare no conflict of interest.

## AUTHOR CONTRIBUTIONS

RLR: designed, analysed data and drafted the manuscript; TG and CO: data collection and contribution contributed to the manuscript; SM supervision supervised the study and reviewed the manuscript of study and review of manuscript; DT design designed the study and contributed to of study and contribution to interpretation of results and drafting of manuscript.

## Data Availability

The data that support the findings of this study are available on request from the corresponding author. The data are not publicly available due to privacy or ethical restrictions.
